# The Cartesian Product and Join Graphs on Edge-Version Atom-Bond Connectivity and Geometric Arithmetic Indices

**DOI:** 10.3390/molecules23071731

**Published:** 2018-07-16

**Authors:** Xiujun Zhang, Huiqin Jiang, Jia-Bao Liu, Zehui Shao

**Affiliations:** 1 Key Laboratory of Pattern Recognition and Intelligent Information Processing, Institutions of Higher Education of Sichuan Province, Chengdu University, Chengdu 610106, China; woodszhang@cdu.edu.cn (X.Z.); hq.jiang@hotmail.com (H.J.); 2 School of Mathematics and Physics, Anhui Jianzhu University, Hefei 230601, China; liujiabaoad@163.com; 3 Institute of Computing Science and Technology, Guangzhou University, Guangzhou 510006, China

**Keywords:** line graph, Cartesian product graph, join graph, atom-bond connectivity index, geometric arithmetic index

## Abstract

The Cartesian product and join are two classical operations in graphs. Let $d_{L(G)}(e)$ be the degree of a vertex e in line graph L(G) of a graph G. The edge versions of atom-bond connectivity ($ABC_{e}$) and geometric arithmetic ($GA_{e}$) indices of *G* are defined as $\sum_{ef\in E(L(G))}\sqrt{\frac{d_{L(G)}(e)+d_{L(G)}(f)-2}{d_{L(G)}(e)\times d_{L(G)}(f)}}$ and $\sum_{ef\in E(L(G))}\frac{2\sqrt{d_{L(G)}(e)\times d_{L(G)}(f)}}{d_{L(G)}(e)+d_{L(G)}(f)}$, respectively. In this paper, $ABC_{e}$ and $GA_{e}$ indices for certain Cartesian product graphs (such as $P_{n}□P_{m}$, $P_{n}□C_{m}$ and $P_{n}□S_{m}$) are obtained. In addition, $ABC_{e}$ and $GA_{e}$ indices of certain join graphs (such as $C_{m}+P_{n}+S_{r}$, $P_{m}+P_{n}+P_{r}$, $C_{m}+C_{n}+C_{r}$ and $S_{m}+S_{n}+S_{r}$) are deduced. Our results enrich and revise some known results.

## 1. Introduction

The invariants based on the distance or degree of vertices in molecules are called topological indices. In theoretical chemistry, physics and graph theory, topological indices are the molecular descriptors that describe the structures of chemical compounds, and they help us to predict certain physico-chemical properties. The first topological index, Wiener index, was published in 1947 [1], and the edge version of the Wiener index was proposed by Iranmanesh et al. in 2009 [2]. Because the important effects of the topological indices are proved in chemical research, more and more topological indices are studied, including the classical atom-bond connectivity index and the geometric arithmetic index.

Let G be a simple connected graph. Denote by V(G) and E(G) the vertex set and edge set of G, respectively. Let $P_{n}$, $C_{n}$, $K_{n}$ and $S_{n}$ be a path, a cycle, a complete graph and a star, respectively, on n vertices. e=uv represents edge-connecting vertices u and v. N(v) is an open neighborhood of vertex v, i.e., N(v)={u|uv∈E(G)}. Denote by $d_{G}(v)$ (simply d(v)) the degree of vertex v of graph G, i.e., d(v)=|N(v)|. Let L(G) or $G^{L}$ be the line graph of G such that each vertex of L(G) represents an edge of G and two vertices of L(G) are adjacent if and only if their corresponding edges share a common endpoint in G [3]. It is known that the line graph L(G) of any graph G is claw-free. Denote by $d_{L(G)}(e)$ the degree of edge e in G, which is the number of edges sharing a common endpoint with edge e in G, or the degree of vertex e in L(G). We denote by $E_{n,m}$ (or $E_{n,m}^{L}$) the set of edges uv with degrees n and m of end vertices u and v in G (or in $G^{L}$), i.e., $E_{n,m}=\{uv|\{n,m\}=\{d(u),d(v)\},$ u∈G,v∈G} or $E_{n,m}^{L}=\{uv|\{n,m\}=\{d(u),d(v)\},u\in L(G),v\in L(G)\}$. The distance $d_{G}(u,v)$ (or d(u,v) for short) between u and v in G is the length of a shortest u−v path.

The atom-bond connectivity (ABC) index was proposed by Estrada et al. in 1998 [4]. The ABC index is defined as:

(1) $$ABC(G)=\sum_{uv\in E(G)}\sqrt{\frac{d_{G}(u)+d_{G}(v)-2}{d_{G}(u)\times d_{G}(v)}}$$

where $d_{G}(u)$ and $d_{G}(v)$ are the degrees of the vertices u and v in G. Meanwhile, the edge version of the *ABC* index is:

(2) $$ABC_{e}(G)=\sum_{ef\in E(L(G))}\sqrt{\frac{d_{L(G)}(e)+d_{L(G)}(f)-2}{d_{L(G)}(e)\times d_{L(G)}(f)}}$$

where $d_{L(G)}(e)$ and $d_{L(G)}(f)$ are the degrees of the edges e and f, respectively, in G. The recent research on edge version *ABC* index can be referred to Gao et al. [5].

The geometric arithmetic (*GA*) index was proposed by Vukicevic and Furthla in 2009 [6]. The *GA* index is defined as

(3) $$GA(G)=\sum_{uv\in E(G)}\frac{2\sqrt{d_{G}(u)d_{G}(v)}}{d_{G}(u)+d_{G}(v)}$$

The edge version of the *GA* index was proposed by Mahmiani et al. [7] and is

(4) $$GA_{e}(G)=\sum_{ef\in E(L(G))}\frac{2\sqrt{d_{L(G)}(e)d_{L(G)}(f)}}{d_{L(G)}(e)+d_{L(G)}(f)}$$

Recent research on the edge-version *GA* index can be referred to the articles [5,8,9,10,11,12,13,14,15,16]. In addition, Das [17] obtained the upper and lower bounds of the *ABC* index of trees. Furtula et al. [18] found the chemical trees with extremal *ABC* values. Fath-Tabar et al. [19] obtained some inequalities for the *ABC* index of a series of graph operations. Chen et al. [20] obtained some upper bounds for the *ABC* index of graphs with given vertex connectivity. Das and Trinajstić [21] compared the *GA* and *ABC* indices for chemical trees and molecular graphs. Xing et al. [22] gave the upper bound for the *ABC* index of trees with perfect matching and characterized the unique extremal tree.

Based on the results, $ABC_{e}$ and $GA_{e}$ indices for certain Cartesian product graphs (such as $P_{n}□P_{m}$, $P_{n}□C_{m}$ and $P_{n}□S_{m}$) are obtained. In addition, $ABC_{e}$ and $GA_{e}$ indices of certain join graphs (such as $C_{m}+P_{n}+S_{r}$, $P_{m}+P_{n}+P_{r}$, $C_{m}+C_{n}+C_{r}$ and $S_{m}+S_{n}+S_{r}$) are deduced. Our results extend and enrich some known results [5,23,24].

## 2. Main Results

It is known that the Cartesian product and join operation are very complicated. In this section, we present these two classical type of graphs.

### 2.1. Cartesian Product Graphs

**Figure 1:**
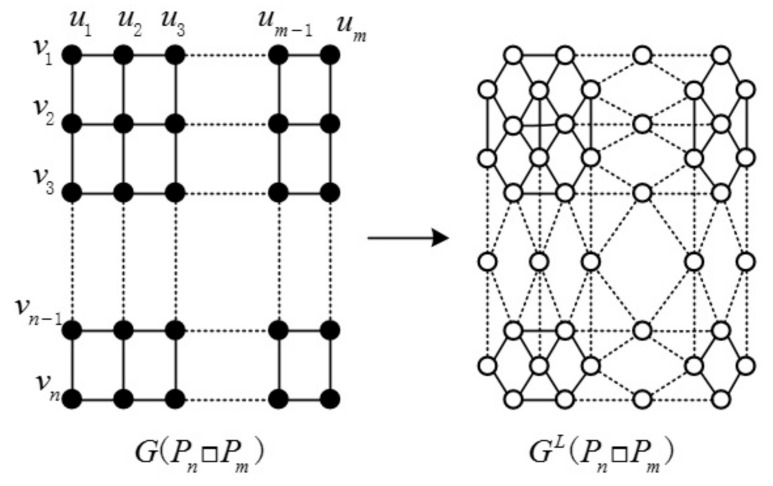
$P_{n}□P_{m}$ and the line graph of $P_{n}□P_{m}$.

In graph theory, the Cartesian product G□H of graphs G and H is a graph such that the vertex set of G□H is the Cartesian product V(G)×V(H); and any two vertices ($u,u^{\prime }$) and ($v,v^{\prime }$) are adjacent in G□H if and only if either u=v and $u^{\prime }$ are adjacent with $v^{\prime }$ in H or $u^{\prime }=v^{\prime }$ and u are adjacent with v in G. The graph $P_{n}□P_{m}$ and the line graph of $P_{n}□P_{m}$ are illustrated in Figure 1. In the following, we discuss the edge-version *ABC* and *GA* indices of some Cartesian product graphs.

**Theorem 1.**  *If* n,m≥4 *, then*

(5) $$\begin{array}{ll} ABC_{e}(P_{n}□P_{m}) & =\frac{1}{2}\sqrt{\frac{3}{2}}(2n+2m-16)+\frac{1}{2}\sqrt{\frac{7}{5}}(4n+4m-24)+\sqrt{\frac{3}{10}}(6n+6m-32)\\ & +\frac{\sqrt{10}}{6}(6nm-18n-18m+52)+\frac{8\sqrt{2}}{5}+4\sqrt{\frac{5}{3}}+8\sqrt{\frac{2}{5}}+\frac{8}{3}; \end{array}$$

(6) $$\begin{array}{ll} GA_{e}(P_{n}□P_{m}) & =6nm-16n-16m+\frac{4\sqrt{5}}{9}(4n+4m-24)+\frac{2\sqrt{30}}{11}(6n+6m-32)\\ & +44+\frac{16\sqrt{12}}{7}+2\sqrt{15}. \end{array}$$

**Proof.**  Let $G=P_{n}□P_{m}$, we have L(G) has 6nm−6n−6m+4 edges. Moreover, $|E_{3,3}^{L}|=4$, $|E_{3,4}^{L}|=8$, $|E_{3,5}^{L}|=8$, $|E_{4,4}^{L}|=2n+2m-16$, $|E_{4,5}^{L}|=4n+4m-24$, $|E_{5,5}^{L}|=4$, $|E_{5,6}^{L}|=6n+6m-32$ and $|E_{6,6}^{L}|=6nm-18n-18m+52$.

(7) $$\begin{array}{ll} ABC_{e}(P_{n}□P_{m}) & =(4)(\sqrt{\frac{3+3-2}{3\times 3}})+(8)(\sqrt{\frac{3+4-2}{3\times 4}})+(8)(\sqrt{\frac{3+5-2}{3\times 5}})\\ & +(2n+2m-16)(\sqrt{\frac{4+4-2}{4\times 4}})\\ & +(4n+4m-24)(\sqrt{\frac{4+5-2}{4\times 5}})+(4)(\sqrt{\frac{5+5-2}{5\times 5}})\\ & +(6n+6m-32)(\sqrt{\frac{5+6-2}{5\times 6}})\\ & +(6nm-18n-18m+52)(\sqrt{\frac{6+6-2}{6\times 6}})\\ & =\frac{1}{2}\sqrt{\frac{3}{2}}(2n+2m-16)+\frac{1}{2}\sqrt{\frac{7}{5}}(4n+4m-24)\\ & +\sqrt{\frac{3}{10}}(6n+6m-32)+\frac{\sqrt{10}}{6}(6nm-18n-18m+52)\\ & +\frac{8\sqrt{2}}{5}+4\sqrt{\frac{5}{3}}+8\sqrt{\frac{2}{5}}+\frac{8}{3}; \end{array}$$

(8) $$\begin{array}{ll} GA_{e}(P_{n}□P_{m}) & =(4)(\frac{2\sqrt{3\times 3}}{3+3})+(8)(\frac{2\sqrt{3\times 4}}{3+4})+(8)(\frac{2\sqrt{3\times 5}}{3+5})\\ & +(2n+2m-16)(\frac{2\sqrt{4\times 4}}{4+4})\\ & +(4n+4m-24)(\frac{2\sqrt{4\times 5}}{4+5})+(4)(\frac{2\sqrt{5\times 5}}{5+5})\\ & +(6n+6m-32)(\frac{2\sqrt{5\times 6}}{5+6})\\ & +(6nm-18n-18m+52)(\frac{2\sqrt{6\times 6}}{6+6})\\ & =6nm-16n-16m+\frac{4\sqrt{5}}{9}(4n+4m-24)\\ & +\frac{2\sqrt{30}}{11}(6n+6m-32)+44+\frac{16\sqrt{12}}{7}+2\sqrt{15}. \end{array}$$

By now, the proof is complete.

**Theorem 2.**  *If* n≥4,m≥3 *, then*

(9) $$ABC_{e}(P_{n}□C_{m})=\sqrt{10}nm+(\frac{\sqrt{6}}{2}+2\sqrt{\frac{7}{5}}+\frac{3\sqrt{30}}{5}-\frac{9\sqrt{10}}{3})m$$

(10) $$GA_{e}(P_{n}□C_{m})=6nm+(2+\frac{16\sqrt{5}}{9}+\frac{12\sqrt{30}}{11}-18)m$$

**Figure 2:**
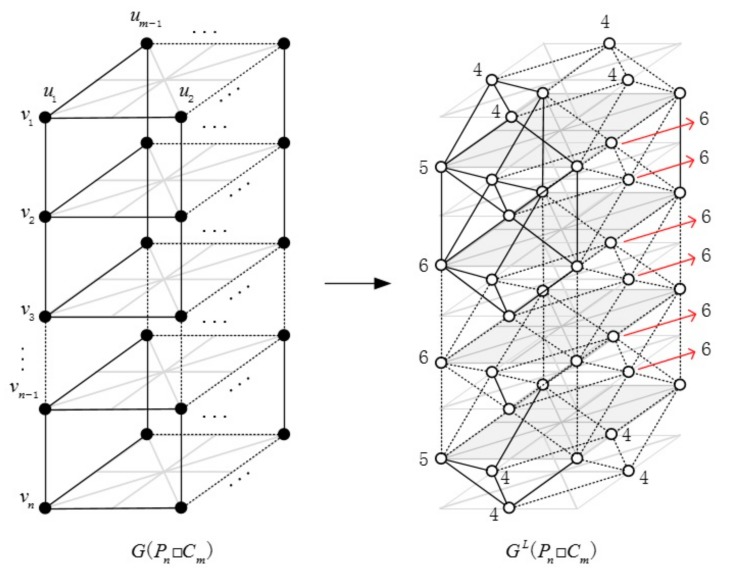
$G(P_{n}□C_{m})$ and $G^{L}(P_{n}□C_{m})$.

**Proof.**  Let $G=P_{n}□C_{m}$, we have L(G) has 6nm−6m edges. Moreover, $|E_{4,4}^{L}|=2m$, $|E_{4,5}^{L}|=4m$, $|E_{5,6}^{L}|=6m$ and $|E_{6,6}^{L}|=6nm-18m$. In Figure 2, the degrees of vertices in line graph $G^{L}(P_{n}□C_{m})$ are displayed near the corresponding vertices.

(11) $$\begin{array}{ll} ABC_{e}(P_{n}□C_{m}) & =(2m)(\sqrt{\frac{4+4-2}{4\times 4}})+(4m)(\sqrt{\frac{4+5-2}{4\times 5}})\\ & +(6m)(\sqrt{\frac{5+6-2}{5\times 6}})+(6nm-18m)(\sqrt{\frac{6+6-2}{6\times 6}})\\ & =\sqrt{10}nm+(\frac{\sqrt{6}}{2}+2\sqrt{\frac{7}{5}}+\frac{3\sqrt{30}}{5}-\frac{9\sqrt{10}}{3})m; \end{array}$$

(12) $$\begin{array}{ll} GA_{e}(P_{n}□C_{m}) & =(2m)(\frac{2\sqrt{4\times 4}}{4+4})+(4m)(\frac{2\sqrt{4\times 5}}{4+5})+(6m)(\frac{2\sqrt{5\times 6}}{5+6})\\ & +(6nm-18m)(\frac{2\sqrt{6\times 6}}{6+6})\\ & =6nm+(2+\frac{16\sqrt{5}}{9}+\frac{12\sqrt{30}}{11}-18)m. \end{array}$$

In the end, the proof is complete.

**Theorem 3.**  *If* n≥5,m≥1 *, then*

(13) $$\begin{array}{ll} ABC_{e}(P_{n}□S_{m}) & =\frac{\left(n-2)(m-1)(m-2\right)}{2(m+2)}\sqrt{2m+2}\\ & +(n-3)(m-1)(\sqrt{\frac{m+4}{m+2}}+2\sqrt{\frac{3}{2(m+2)}})\\ & +2(m-1)(\sqrt{\frac{m+3}{3(m+2)}}+\sqrt{\frac{3m-1}{\left(m+2)(2m-1\right)}}+\sqrt{\frac{m+1}{3m}}\\ & +\sqrt{\frac{3m-3}{m(2m-1)}})+\frac{\left(m-1)(m-2\right)}{m}\sqrt{2m-2}\\ & +\frac{1}{4}(m-1)(n-4)\sqrt{6}+(m-1)\sqrt{\frac{5}{3}}\\ & +\frac{n-4}{2m}\sqrt{4m-2}+2\sqrt{\frac{4m-3}{2m(2m-1)}}; \end{array}$$

(14) $$\begin{array}{ll} GA_{e}(P_{n}□S_{m}) & =\frac{\left(n-2)(m-1)(m-2\right)}{2}+8(n-3)(m-1)\frac{\sqrt{\left(m+2\right)}}{m+6}\\ & +4(m-1)\frac{\sqrt{3(m+2)}}{m+5}+4(n-3)(m-1)\frac{\sqrt{2m(m+2)}}{3m+2}\\ & +4(m-1)\frac{\sqrt{\left(m+2)(2m-1\right)}}{3m+1}+(m-1)(m-2)\\ & +4(m-1)\frac{\sqrt{3m}}{m+3}+4(m-1)\frac{\sqrt{m(2m-1)}}{3m-1}\\ & +(m-1)(n-4)+8(m-1)\frac{\sqrt{3}}{7}\\ & +(n-4)+4\frac{\sqrt{2m(2m-1)}}{4m-1}. \end{array}$$

**Figure 3:**
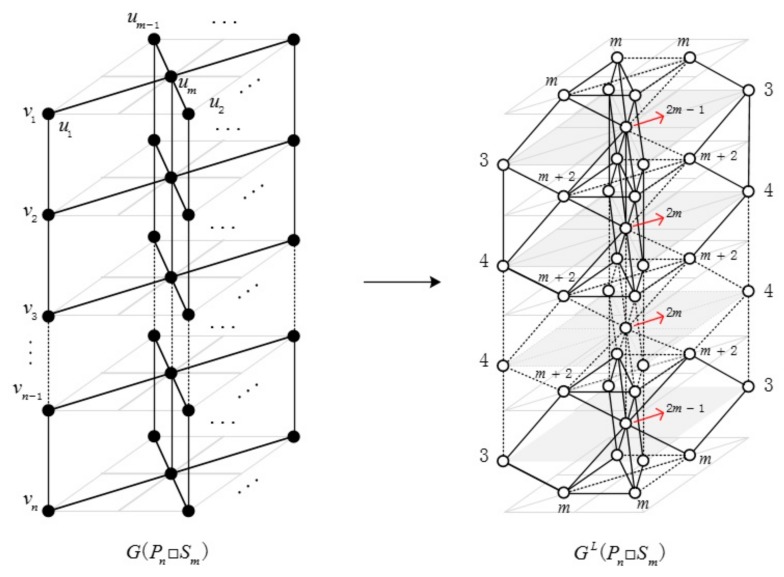
$G(P_{n}□S_{m})$ and $G^{L}(P_{n}□S_{m})$.

**Proof.**  Let $G=P_{n}□S_{m}$, we have L(G) has $\frac{1}{2}(m^{2}n+m(7n-10)-8n+8)$ edges. Moreover, $|E_{m+2,m+2}^{L}|=\frac{\left(n-2)(m-1)(m-2\right)}{2}$, $\left|E_{m+2,4}^{L}|=2(n-3)(m-1\right)$, $\left|E_{m+2,3}^{L}|=2(m-1\right)$, $\left|E_{m+2,2m}^{L}|=2(n-3)(m-1\right)$, $\left|E_{m+2,2m-1}^{L}|=2(m-1\right)$, $\left|E_{m,m}^{L}|=(m-1)(m-2\right)$, $\left|E_{m,3}^{L}|=2(m-1\right)$, $\left|E_{m,2m-1}^{L}|=2(m-1\right)$, $\left|E_{4,4}^{L}|=(m-1)(n-4\right)$, $\left|E_{3,4}^{L}|=2(m-1\right)$, $\left|E_{2m,2m}^{L}|=(n-4\right)$ and $|E_{2m-1,2m}^{L}|=2$. In Figure 3, the degrees of vertices in line graph $G^{L}(P_{n}□S_{m})$ are displayed near by the corresponding vertices.

(15) $$\begin{array}{ll} ABC_{e}(P_{n}□S_{m}) & =\frac{\left(n-2)(m-1)(m-2\right)}{2}(\sqrt{\frac{m+2+m+2-2}{\left(m+2)\times (m+2\right)}})\\ & +2(n-3)(m-1)(\sqrt{\frac{m+2+4-2}{(m+2)\times 4}})\\ & +2(m-1)(\sqrt{\frac{m+2+3-2}{(m+2)\times 3}})\\ & +2(n-3)(m-1)(\sqrt{\frac{m+2+2m-2}{(m+2)\times 2m}})\\ & +2(m-1)(\sqrt{\frac{m+2+2m-1-2}{\left(m+2)\times (2m-1\right)}})\\ & +(m-1)(m-2)(\sqrt{\frac{m+m-2}{m\times m}})\\ & +2(m-1)(\sqrt{\frac{m+3-2}{m\times 3}})+2(m-1)(\sqrt{\frac{m+2m-1-2}{m\times (2m-1)}})\\ & +(m-1)(n-4)(\sqrt{\frac{4+4-2}{4\times 4}})+2(m-1)(\sqrt{\frac{3+4-2}{3\times 4}})\\ & +(n-4)(\sqrt{\frac{2m+2m-2}{2m\times 2m}})+2(\sqrt{\frac{2m-1+2m-2}{(2m-1)\times 2m}})\\ & =\frac{\left(n-2)(m-1)(m-2\right)}{2(m+2)}\sqrt{2m+2}\\ & +(n-3)(m-1)(\sqrt{\frac{m+4}{m+2}}+2\sqrt{\frac{3}{2(m+2)}})\\ & +2(m-1)(\sqrt{\frac{m+3}{3(m+2)}}+\sqrt{\frac{3m-1}{\left(m+2)(2m-1\right)}}\\ & +\sqrt{\frac{m+1}{3m}}+\sqrt{\frac{3m-3}{m(2m-1)}})\\ & +\frac{\left(m-1)(m-2\right)}{m}\sqrt{2m-2}\\ & +\frac{1}{4}(m-1)(n-4)\sqrt{6}+(m-1)\sqrt{\frac{5}{3}}\\ & +\frac{n-4}{2m}\sqrt{4m-2}+2\sqrt{\frac{4m-3}{2m(2m-1)}}; \end{array}$$

(16) $$\begin{array}{ll} GA_{e}(P_{n}□S_{m}) & =\frac{\left(n-2)(m-1)(m-2\right)}{2}(\frac{2\sqrt{\left(m+2)\times (m+2\right)}}{m+2+m+2})\\ & +2(n-3)(m-1)(\frac{2\sqrt{(m+2)\times 4}}{m+2+4})\\ & +2(m-1)(\frac{2\sqrt{(m+2)\times 3}}{m+2+3})\\ & +2(n-3)(m-1)(\frac{2\sqrt{(m+2)\times 2m}}{m+2+2m})\\ & +2(m-1)(\frac{2\sqrt{\left(m+2)\times (2m-1\right)}}{m+2+2m-1})\\ & +(m-1)(m-2)(\frac{2\sqrt{m\times m}}{m+m})\\ & +2(m-1)(\frac{2\sqrt{m\times 3}}{m+3})+2(m-1)(\frac{2\sqrt{m\times (2m-1)}}{m+2m-1})\\ & +(m-1)(n-4)(\frac{2\sqrt{4\times 4}}{4+4})+2(m-1)(\frac{2\sqrt{3\times 4}}{3+4})\\ & +(n-4)(\frac{2\sqrt{2m\times 2m}}{2m+2m})+2(\frac{2\sqrt{\left(2m-1)\times (2m\right)}}{2m-1+2m})\\ & =\frac{\left(n-2)(m-1)(m-2\right)}{2}+8(n-3)(m-1)\frac{\sqrt{\left(m+2\right)}}{m+6}\\ & +4(m-1)\frac{\sqrt{3(m+2)}}{m+5}+4(n-3)(m-1)\frac{\sqrt{2m(m+2)}}{3m+2}\\ & +4(m-1)\frac{\sqrt{\left(m+2)(2m-1\right)}}{3m+1}+(m-1)(m-2)\\ & +4(m-1)\frac{\sqrt{3m}}{m+3}+4(m-1)\frac{\sqrt{m(2m-1)}}{3m-1}\\ & +(m-1)(n-4)+8(m-1)\frac{\sqrt{3}}{7}\\ & +(n-4)+4\frac{\sqrt{2m(2m-1)}}{4m-1}. \end{array}$$

Until now, the proof is complete.

### 2.2. Join Graph

The results of $ABC_{e}$ and $GA_{e}$ indices of $P_{n}$, $S_{n}$, $K_{n}$ and $C_{n}$, which were first established by [7], as well as the $ABC_{e}$ and $GA_{e}$ indices of some join graphs, such as $P_{n}+C_{m}$, $P_{n}+S_{m}$, $C_{m}+P_{n}+C_{m}$, $S_{m}+P_{n}+S_{m}$ and $C_{m}+P_{n}+S_{r}$, created by $P_{n}$, $C_{n}$ and $S_{n}$ were obtained by [5]. However, there are some problems in the calculation of the $ABC_{e}$ and $GA_{e}$ indices of join graph $C_{m}+P_{n}+S_{r}$ in [5].

**Figure 4:**
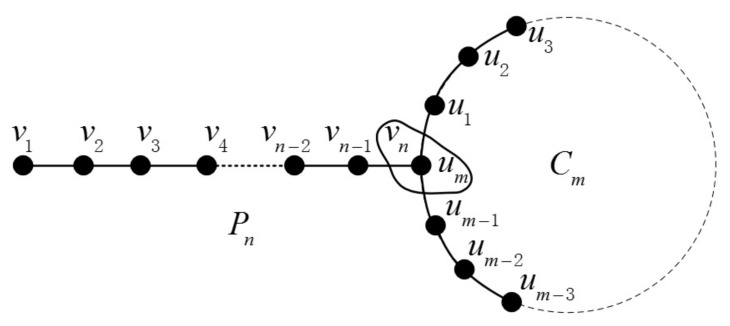
The join graph of $P_{n}+C_{m}$.

**Figure 5:**
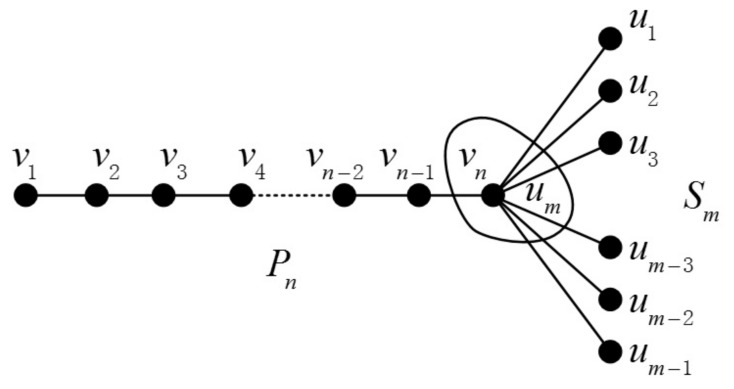
The join graph of $P_{n}+S_{m}$.

The join graph operation’s definition is given as follows: If we are given two graphs G and H and two vertices $v_{i}\in V(G)$, $u_{j}\in V(H)$, the join graph is obtained by merging $v_{i}$ and $u_{j}$ into one vertex. The certain join graphs $P_{n}+C_{m}$ and $P_{n}+S_{m}$ are illustrated in Figure 4 and Figure 5, respectively.

Theorem A is stated in [5]. However, the result is not correct. In this paper, we correct the result of Theorem A and restate it in Theorem 4 as follows:

**Theorem A.**  *If* n,r≥4,m≥3 *, then*

(17) $$ABC_{e}(C_{m}+P_{n}+S_{r})=\frac{r-2}{2}\sqrt{2r-4}+(r-1)\sqrt{\frac{2r-3}{r(r-1)}}+\frac{\sqrt{2}}{2}(n+m-3)+2$$

(18) $$GA_{e}(C_{m}+P_{n}+S_{r})=\frac{2\sqrt{2(r-1)}}{r+1}+(r-1)(\frac{r-2}{2}+\frac{2\sqrt{r(r-1)}}{2r-1})+n+m+\frac{6\sqrt{6}}{5}-4$$

**Figure 6:**
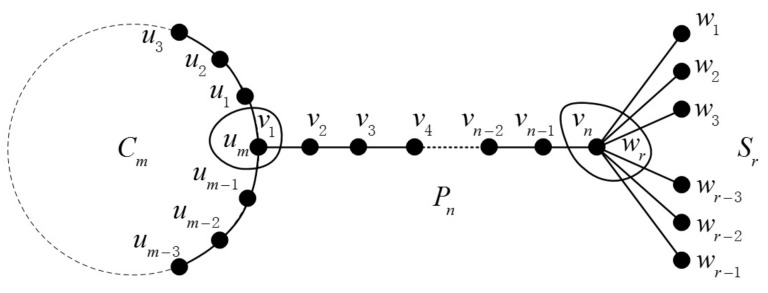
The join graph of $C_{m}+P_{n}+S_{r}$.

The join graph of $C_{m}+P_{n}+S_{r}$ is illustrated in Figure 6. It can be seen that $d_{L(G)}(v_{n-2}v_{n-1})$ is 2 and $d_{L(G)}(v_{n-1}v_{n})$ is r in $C_{m}+P_{n}+S_{r}$, so we have one edge of types $d_{L(G)}(e)=2$ and $d_{L(G)}(f)=r$ in $G^{L}(C_{m}+P_{n}+S_{r})$.

**Theorem 4.**  *If* n≥4,r≥4,m≥3 *, then we have*

(19) $$ABC_{e}(C_{m}+P_{n}+S_{r})=\frac{r-2}{2}\sqrt{2r-4}+(r-1)\sqrt{\frac{2r-3}{r(r-1)}}+\frac{\sqrt{2}}{2}(n+m-3)+2$$

(20) $$GA_{e}(C_{m}+P_{n}+S_{r})=\frac{2\sqrt{2r}}{r+2}+(r-1)(\frac{r-2}{2}+\frac{2\sqrt{r(r-1)}}{2r-1})+n+m+\frac{6\sqrt{6}}{5}-4$$

**Proof.**  Let $G=C_{m}+P_{n}+S_{r}$, we have $|E_{2,2}^{L}|=n+m-7$, $|E_{2,3}^{L}|=3$, $|E_{2,r}^{L}|=1$, $|E_{3,3}^{L}|=3$, $|E_{r-1,r-1}^{L}|=\frac{\left(r-1)(r-2\right)}{2}$ and $|E_{r-1,r}^{L}|=r-1$.

(21) $$\begin{array}{ll} ABC_{e}(C_{m}+P_{n}+S_{r}) & =(n+m-7)ABC_{e}(E_{2,2}^{L})+(3)ABC_{e}^{L}(E_{2,3})\\ & +(1)ABC_{e}(E_{2,r}^{L})+(3)ABC_{e}(E_{3,3}^{L})\\ & +\frac{\left(r-1)(r-2\right)}{2}ABC_{e}(E_{r-1,r-1}^{L})\\ & +(r-1)ABC_{e}(E_{r-1,r}^{L})\\ & =(n+m-7)(\sqrt{\frac{2+2-2}{2\times 2}})+(3)(\sqrt{\frac{2+3-2}{2\times 3}})\\ & +(1)(\sqrt{\frac{2+r-2}{2\times r}})+(3)(\sqrt{\frac{3+3-2}{3\times 3}})\\ & +\frac{\left(r-1)(r-2\right)}{2}(\sqrt{\frac{(r-1)+(r-1)-2}{\left(r-1)\times (r-1\right)}})\\ & +(r-1)(\sqrt{\frac{(r-1)+r-2}{(r-1)\times r}})\\ & =\frac{r-2}{2}\sqrt{2r-4}+(r-1)\sqrt{\frac{2r-3}{r(r-1)}}\\ & +\frac{\sqrt{2}}{2}(n+m-3)+2. \end{array}$$

Remark: The result of $ABC_{e}(C_{m}+P_{n}+S_{r})$ is the same as that of [5], only because the $ABC_{e}(E_{2,r-1}^{L})=ABC_{e}(E_{2,r}^{L})$. We must note $GA_{e}(E_{2,r-1}^{L})\neq GA_{e}(E_{2,r}^{L})$.

(22) $$\begin{array}{ll} GA_{e}(C_{m}+P_{n}+S_{r}) & =(n+m-7)GA_{e}(E_{2,2}^{L})+(3)GA_{e}(E_{2,3}^{L})+(1)GA_{e}(E_{2,r}^{L})\\ & +(3)GA_{e}(E_{3,3}^{L})+\frac{\left(r-1)(r-2\right)}{2}GA_{e}(E_{r-1,r}^{L})\\ & =(n+m-7)(\frac{2\sqrt{2\times 2}}{2+2})+(3)(\frac{2\sqrt{2\times 3}}{2+3})\\ & +(1)(\frac{2\sqrt{2\times r}}{2+r})+(3)(\frac{2\sqrt{3\times 3}}{3+3})\\ & +\frac{\left(r-1)(r-2\right)}{2}(\frac{2\sqrt{\left(r-1)\times (r-1\right)}}{\left(r-1)+(r-1\right)})\\ & +(r-1)(\frac{2\sqrt{(r-1)\times r}}{(r-1)+r})\\ & =\frac{2\sqrt{2r}}{r+2}+(r-1)(\frac{r-2}{2}+\frac{2\sqrt{r(r-1)}}{2r-1})\\ & +n+m+\frac{6\sqrt{6}}{5}-4. \end{array}$$

Now the proof is complete.

**Figure 7:**
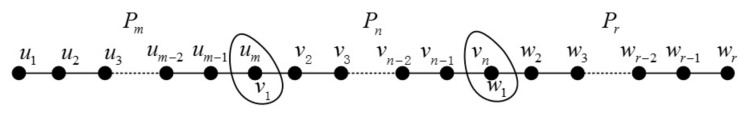
The join graph of $P_{m}+P_{n}+P_{r}$.

**Theorem 5.**  *If*m≥2,n≥2,r≥2*and*$P_{m}+P_{n}+P_{r}$*be the join graphs depicted in Figure 7, then*

(23) $$ABC_{e}(P_{m}+P_{n}+P_{r})=\frac{\sqrt{2}}{2}(m+n+r-4)$$

(24) $$GA_{e}(P_{m}+P_{n}+P_{r})=m+n+r-6+\frac{4}{3}\sqrt{2}$$.

**Proof.**  Let $G=P_{m}+P_{n}+P_{r}$, we have $|E_{2,2}^{L}|=m+n+r-6$ and $|E_{1,2}^{L}|=2$.

(25) $$\begin{array}{ll} ABC_{e}(P_{m}+P_{n}+P_{r}) & =(m+n+r-6)ABC_{e}(E_{2,2}^{L})+2ABC_{e}(E_{1,2}^{L})\\ & =(m+n+r-6)(\sqrt{\frac{2+2-2}{2\times 2}})+2(\sqrt{\frac{1+2-2}{1\times 2}})\\ & =\frac{\sqrt{2}}{2}(m+n+r-4). \end{array}$$

(26) $$\begin{array}{ll} GA_{e}(P_{m}+P_{n}+P_{r}) & =(m+n+r-6)GA_{e}(E_{2,2}^{L})+2GA_{e}(E_{1,2}^{L})\\ & =(m+n+r-6)(\frac{2\sqrt{2\times 2}}{2+2})+2(\frac{2\sqrt{1\times 2}}{1+2})\\ & =m+n+r-6+\frac{4}{3}\sqrt{2}. \end{array}$$

Now the proof is complete.

**Figure 8:**
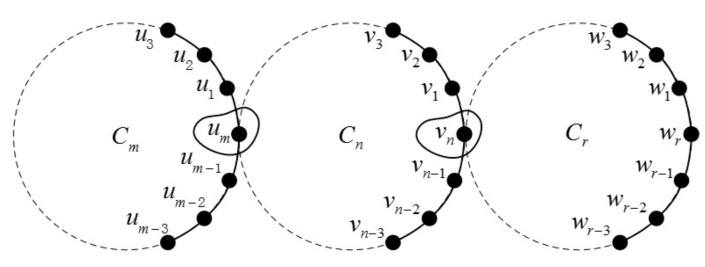
The join graph of $C_{m}+C_{n}+C_{r}$.

**Theorem 6.**  *Let*m≥3,r≥3,n≥6*and*$C_{m}+C_{n}+C_{r}$*be the join graphs depicted in Figure 8. If*$d(u_{m},v_{n})\geq 3$*, then*

(27) $$ABC_{e}(C_{m}+C_{n}+C_{r})=\frac{\sqrt{2}}{2}(m+n+r)-2\sqrt{2}+3\sqrt{6}$$

(28) $$GA_{e}(C_{m}+C_{n}+C_{r})=m+n+r+\frac{16\sqrt{2}}{3}$$.

**Proof.**  Let $G=C_{m}+C_{n}+C_{r}$, we have $|E_{2,2}^{L}|=m+n+r-12$, $|E_{2,4}^{L}|=8$ and $|E_{4,4}^{L}|=12$.

(29) $$\begin{array}{ll} ABC_{e}(C_{m}+C_{n}+C_{r}) & =(m+n+r-12)ABC_{e}(E_{2,2}^{L})\\ & +8ABC_{e}(E_{2,4}^{L})+12ABC_{e}(E_{4,4}^{L})\\ & =(m+n+r-12)(\sqrt{\frac{2+2-2}{2\times 2}})\\ & +8(\sqrt{\frac{2+4-2}{2\times 4}})+12(\sqrt{\frac{4+4-2}{4\times 4}})\\ & =\frac{\sqrt{2}}{2}(m+n+r)-2\sqrt{2}+3\sqrt{6}. \end{array}$$

(30) $$\begin{array}{ll} GA_{e}(P_{m}+P_{n}+P_{r}) & =(m+n+r-12)GA_{e}(E_{2,2}^{L})\\ & +8GA_{e}(E_{2,4}^{L})+12GA_{e}(E_{4,4}^{L})\\ & =(m+n+r-12)(\frac{2\sqrt{2\times 2}}{2+2})\\ & +8(\frac{2\sqrt{2\times 4}}{2+4})+12(\frac{2\sqrt{4\times 4}}{4+4})\\ & =m+n+r+\frac{16\sqrt{2}}{3}. \end{array}$$

Now the proof is complete.

**Figure 9:**
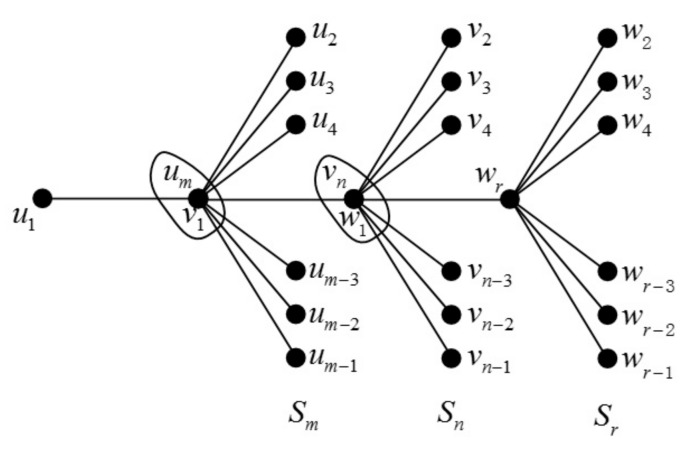
The join graph of $S_{m}+S_{n}+S_{r}$.

**Theorem 7.**  *Let*m≥2,n≥3,r≥3*and*$S_{m}+S_{n}+S_{r}$*be the join graphs depicted in Figure 9; then, we have*

(31) $$\begin{array}{ll} ABC_{e}(S_{m}+S_{n}+S_{r}) & =(m-1)\sqrt{\frac{2m+n-5}{\left(m-1)(m+n-2\right)}}+(n-2)\sqrt{\frac{m+2n-5}{\left(n-1)(m+n-2\right)}}\\ & +(n-2)\sqrt{\frac{2n+r-6}{\left(n-1)(n+r-3\right)}}+(r-2)\sqrt{\frac{n+2r-7}{\left(r-2)(n+r-3\right)}}\\ & +\frac{\left(m-2\right)}{2}\sqrt{2m-4}+\frac{\left(n-2)(n-3\right)}{2(n-1)}\sqrt{2n-4}+\frac{\left(r-3\right)}{2}\sqrt{2r-6}\\ & +\sqrt{\frac{m+2n+r-7}{\left(m+n-2)(n+r-3\right)}}; \end{array}$$

(32) $$\begin{array}{ll} GA_{e}(S_{m}+S_{n}+S_{r}) & =2(m-1)\frac{\sqrt{\left(m-1)(m+n-2\right)}}{2m+n-3}+2(n-2)\frac{\sqrt{\left(n-1)(m+n-2\right)}}{m+2n-3}\\ & +2(n-2)\frac{\sqrt{\left(n-1)(n+r-3\right)}}{2n+r-4}+2(r-2)\frac{\sqrt{\left(r-2)(n+r-3\right)}}{n+2r-5}\\ & +\frac{\left(m-1)(m-2\right)}{2}+\frac{\left(n-2)(n-3\right)}{2}+\frac{\left(r-2)(r-3\right)}{2}\\ & +\frac{2\sqrt{\left(m+n-2)(n+r-3\right)}}{m+2n+r-5}. \end{array}$$.

**Proof.**  Let $G=S_{m}+S_{n}+S_{r}$, we have $|E_{m-1,m+n-2}^{L}|=m-1$, $|E_{n-1,m+n-2}^{L}|=n-2$, $|E_{n-1,n+r-3}^{L}|=n-2$, $|E_{r-2,n+r-3}^{L}|=r-2$, $|E_{m-1,m-1}^{L}|=\frac{\left(m-1)(m-2\right)}{2}$, $|E_{n-1,n-1}^{L}|=\frac{\left(n-2)(n-3\right)}{2}$, $|E_{r-2,r-2}^{L}|=\frac{\left(r-2)(r-3\right)}{2}$ and $|E_{m+n-2,n+r-3}^{L}|=1$.

(33) $$\begin{array}{ll} ABC_{e}(S_{m}+S_{n}+S_{r}) & =(m-1)ABC_{e}(E_{m-1,m+n-2}^{L})\\ & +(n-2)ABC_{e}(E_{n-1,m+n-2}^{L})\\ & +(n-2)ABC_{e}(E_{n-1,n+r-3}^{L})\\ & +(r-2)ABC_{e}(E_{r-2,n+r-3}^{L})\\ & +\frac{\left(m-1)(m-2\right)}{2}ABC_{e}(E_{m-1,m-1}^{L})\\ & +\frac{\left(n-2)(n-3\right)}{2}ABC_{e}(E_{n-1,n-1}^{L})\\ & +\frac{\left(r-2)(r-3\right)}{2}ABC_{e}(E_{r-2,r-2}^{L})\\ & +(1)ABC_{e}(E_{m+n-2,n+r-3}^{L})\\ & =(m-1)\sqrt{\frac{(m-1)+(m+n-2)-2}{\left(m-1)(m+n-2\right)}}\\ & +(n-2)\sqrt{\frac{(n-1)+(m+n-2)-2}{\left(n-1)(m+n-2\right)}}\\ & +(n-2)\sqrt{\frac{(n-1)+(n+r-3)-2}{\left(n-1)(n+r-3\right)}}\\ & +(r-2)\sqrt{\frac{(r-2)+(n+r-3)-2}{\left(r-2)(n+r-3\right)}}\\ & +\frac{\left(m-1)(m-2\right)}{2}\sqrt{\frac{(m-1)+(m-1)-2}{\left(m-1)(m-1\right)}}\\ & +\frac{\left(n-2)(n-3\right)}{2}\sqrt{\frac{(n-1)+(n-1)-2}{\left(n-1)(n-1\right)}}\\ & +\frac{\left(r-2)(r-3\right)}{2}\sqrt{\frac{(r-2)+(r-2)-2}{\left(r-2)(r-2\right)}}\\ & +(1)\sqrt{\frac{(m+n-2)+(n+r-3)-2}{\left(m+n-2)(n+r-3\right)}}\\ & =(m-1)\sqrt{\frac{2m+n-5}{\left(m-1)(m+n-2\right)}}\\ & +(n-2)\sqrt{\frac{m+2n-5}{\left(n-1)(m+n-2\right)}}\\ & +(n-2)\sqrt{\frac{2n+r-6}{\left(n-1)(n+r-3\right)}}\\ & +(r-2)\sqrt{\frac{n+2r-7}{\left(r-2)(n+r-3\right)}}\\ & +\frac{\left(m-2\right)}{2}\sqrt{2m-4}+\frac{\left(n-2)(n-3\right)}{2(n-1)}\sqrt{2n-4}\\ & +\frac{\left(r-3\right)}{2}\sqrt{2r-6}+\sqrt{\frac{m+2n+r-7}{\left(m+n-2)(n+r-3\right)}}. \end{array}$$

(34) $$\begin{array}{ll} GA_{e}(S_{m}+S_{n}+S_{r}) & =(m-1)GA_{e}(E_{m-1,m+n-2}^{L})\\ & +(n-2)GA_{e}(E_{n-1,m+n-2}^{L})\\ & +(n-2)GA_{e}(E_{n-1,n+r-3}^{L})\\ & +(r-2)GA_{e}(E_{r-2,n+r-3}^{L})\\ & +\frac{\left(m-1)(m-2\right)}{2}GA_{e}(E_{m-1,m-1}^{L})\\ & +\frac{\left(n-2)(n-3\right)}{2}GA_{e}(E_{n-1,n-1}^{L})\\ & +\frac{\left(r-2)(r-3\right)}{2}GA_{e}(E_{r-2,r-2}^{L})\\ & +(1)ABC_{e}(E_{m+n-2,n+r-3}^{L})\\ & =(m-1)\frac{2\sqrt{\left(m-1)(m+n-2\right)}}{\left(m-1)+(m+n-2\right)}\\ & +(n-2)\frac{2\sqrt{\left(n-1)(m+n-2\right)}}{\left(n-1)+(m+n-2\right)}\\ & +(n-2)\frac{2\sqrt{\left(n-1)(n+r-3\right)}}{\left(n-1)+(n+r-3\right)}\\ & +(r-2)\frac{2\sqrt{\left(r-2)(n+r-3\right)}}{\left(r-2)+(n+r-3\right)}\\ & +\frac{\left(m-1)(m-2\right)}{2}\frac{2\sqrt{\left(m-1)(m-1\right)}}{\left(m-1)+(m-1\right)}\\ & +\frac{\left(n-2)(n-3\right)}{2}\frac{2\sqrt{\left(n-1)(n-1\right)}}{\left(n-1)+(n-1\right)}\\ & +\frac{\left(r-2)(r-3\right)}{2}\frac{2\sqrt{\left(r-2)(r-2\right)}}{\left(r-2)+(r-2\right)}\\ & +(1)\frac{2\sqrt{\left(m+n-2)(n+r-3\right)}}{\left(m+n-2)+(n+r-3\right)}\\ & =2(m-1)\frac{\sqrt{\left(m-1)(m+n-2\right)}}{2m+n-3}\\ & +2(n-2)\frac{\sqrt{\left(n-1)(m+n-2\right)}}{m+2n-3}\\ & +2(n-2)\frac{\sqrt{\left(n-1)(n+r-3\right)}}{2n+r-4}\\ & +2(r-2)\frac{\sqrt{\left(r-2)(n+r-3\right)}}{n+2r-5}\\ & +\frac{\left(m-1)(m-2\right)}{2}+\frac{\left(n-2)(n-3\right)}{2}\\ & +\frac{\left(r-2)(r-3\right)}{2}\\ & +\frac{2\sqrt{\left(m+n-2)(n+r-3\right)}}{m+2n+r-5}. \end{array}$$

Now the proof is complete.

## 3. Conclusions

The physical and chemical properties of proteins, DNAs and RNAs are very important for human disease and various approaches have been proposed to predict, validate and identify their structures and features [25,26]. Among these, topological indices were proved to be very helpful in testing the chemical properties of new chemical or physical materials such as new drugs or nanomaterials. Topological indices play an important role in studying the topological properties of chemical compounds, especially organic materials i.e., carbon containing molecular structures.

Various topological indices provide a better correlation for certain physico-chemical properties. Hence, the edge version ABC and GA indices for some special Cartesian product graphs and certain join graphs are described by graph structure analysis and a mathematical derivation method in this paper. The results of the current study also have promising prospects for applications in chemical and material engineering. The conclusions we draw here will not work for other classes of indices such as distance-based and distance adjacency-based topological indices. Thus a similar kind of study is needed for other classes of indices which might be a future direction in this area of mathematical chemistry.
